# Supplementary material for: A Humanized Yeast Phenomic Model of Deoxycytidine Kinase to Predict Genetic Buffering of Nucleoside Analog Cytotoxicity
Source: Genes (Basel). 2019 Sep 30;10(10):770. doi: 10.3390/genes10100770 (PMC6826991; doi:10.3390/genes10100770)
Supplement: Supplementary file 1 [file genes-10-00770-s001.zip › Additional_Files/Additional_File7_GOTermHeatmaps.zip!/Additional_File7_GOTermHeatmaps/chromatin_binding.pdf]

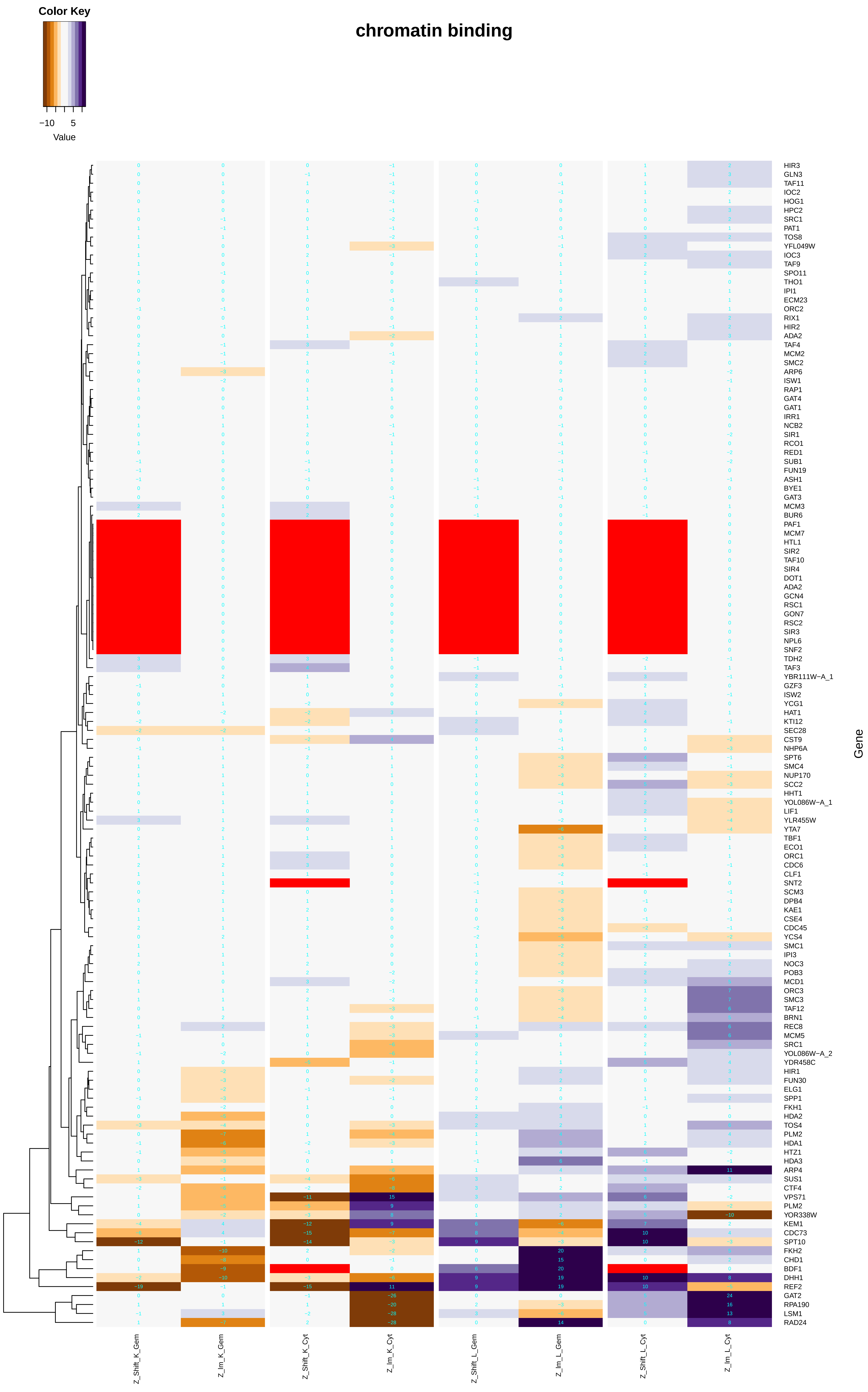

chromatin binding
Color Key
−10
5
Value
Z_Shift_K_Gem
Z_Im_K_Gem
Z_Shift_K_Cyt
Z_Im_K_Cyt
Z_Shift_L_Gem
Z_Im_L_Gem
Z_Shift_L_Cyt
Z_Im_L_Cyt
Gene
HIR3
GLN3
TAF11
IOC2
HOG1
HPC2
SRC1
PAT1
TOS8
YFL049W
IOC3
TAF9
SPO11
THO1
IPI1
ECM23
ORC2
RIX1
HIR2
ADA2
TAF4
MCM2
SMC2
ARP6
ISW1
RAP1
GAT4
GAT1
IRR1
NCB2
SIR1
RCO1
RED1
SUB1
FUN19
ASH1
BYE1
GAT3
MCM3
BUR6
PAF1
MCM7
HTL1
SIR2
TAF10
SIR4
DOT1
ADA2
GCN4
RSC1
GON7
RSC2
SIR3
NPL6
SNF2
TDH2
TAF3
YBR111W−A_1
GZF3
ISW2
YCG1
HAT1
KTI12
SEC28
CST9
NHP6A
SPT6
SMC4
NUP170
SCC2
HHT1
YOL086W−A_1
LIF1
YLR455W
YTA7
TBF1
ECO1
ORC1
CDC6
CLF1
SNT2
SCM3
DPB4
KAE1
CSE4
CDC45
YCS4
SMC1
IPI3
NOC3
POB3
MCD1
ORC3
SMC3
TAF12
BRN1
REC8
MCM5
SRC1
YOL086W−A_2
YDR458C
HIR1
FUN30
ELG1
SPP1
FKH1
HDA2
TOS4
PLM2
HDA1
HTZ1
HDA3
ARP4
SUS1
CTF4
VPS71
PLM2
YOR338W
KEM1
CDC73
SPT10
FKH2
CHD1
BDF1
DHH1
REF2
GAT2
RPA190
LSM1
RAD24

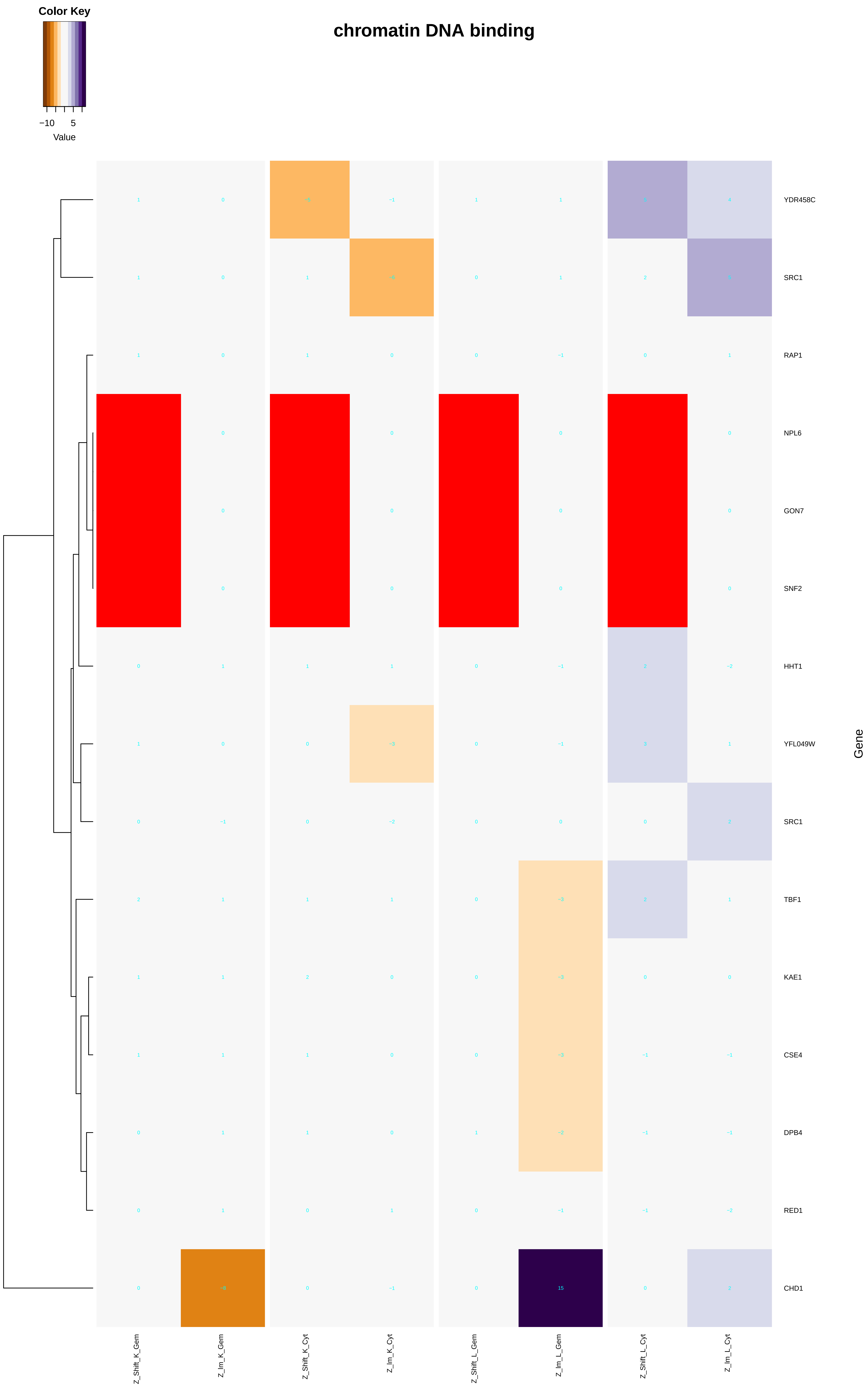

Color Key
−10
5
Value
chromatin DNA binding
YDR458C
SRC1
RAP1
NPL6
GON7
SNF2
HHT1
YFL049W
SRC1
TBF1
KAE1
CSE4
DPB4
RED1
CHD1
Gene
Z_Shift_K_Gem
Z_Im_K_Gem
Z_Shift_K_Cyt
Z_Im_K_Cyt
Z_Shift_L_Gem
Z_Im_L_Gem
Z_Shift_L_Cyt
Z_Im_L_Cyt

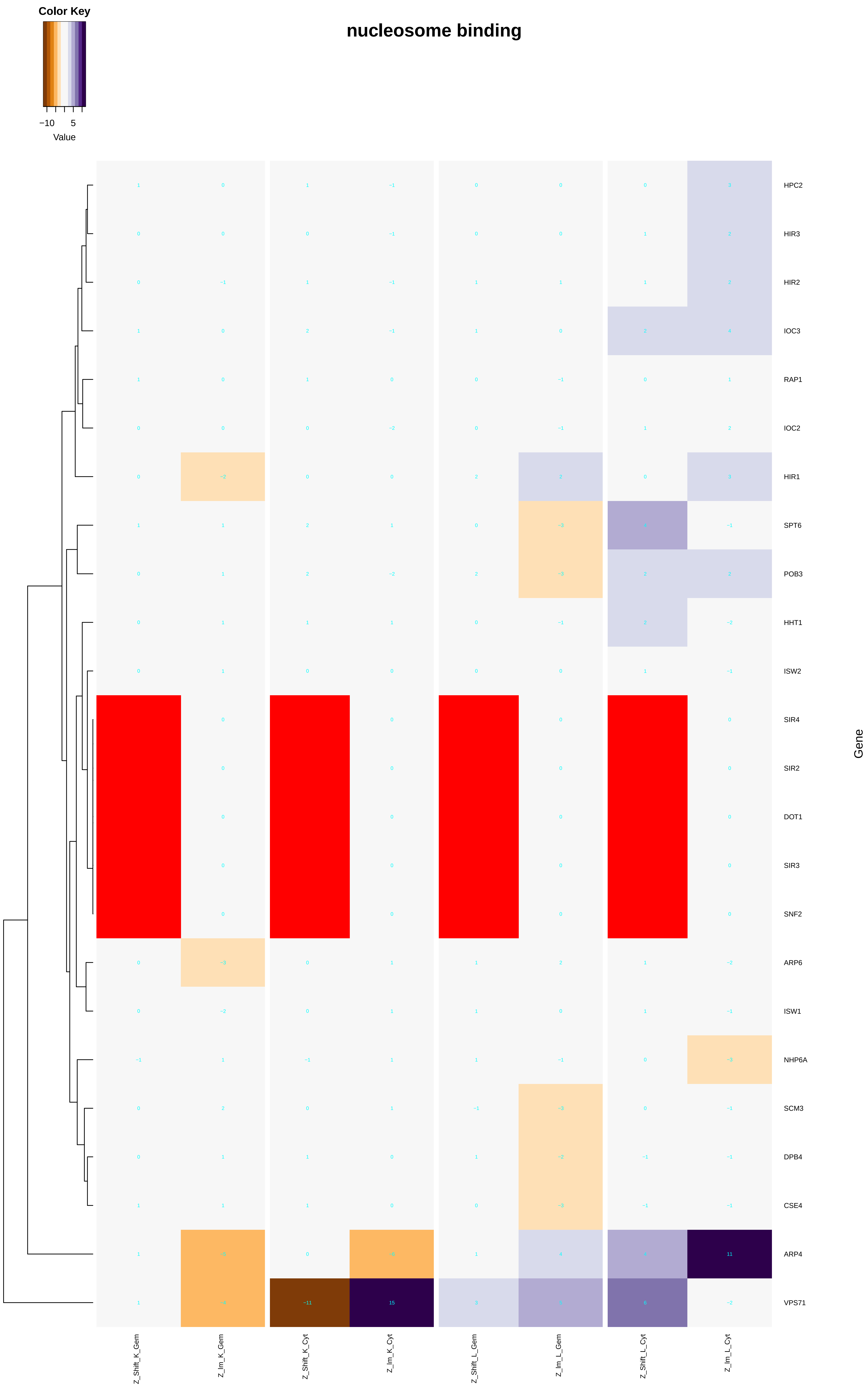

Color Key
−10
5
Value
nucleosome binding
HPC2
HIR3
HIR2
IOC3
RAP1
IOC2
HIR1
SPT6
POB3
HHT1
ISW2
SIR4
SIR2
DOT1
SIR3
SNF2
ARP6
ISW1
NHP6A
SCM3
DPB4
CSE4
ARP4
VPS71
Gene
Z_Shift_K_Gem
Z_Im_K_Gem
Z_Shift_K_Cyt
Z_Im_K_Cyt
Z_Shift_L_Gem
Z_Im_L_Gem
Z_Shift_L_Cyt
Z_Im_L_Cyt

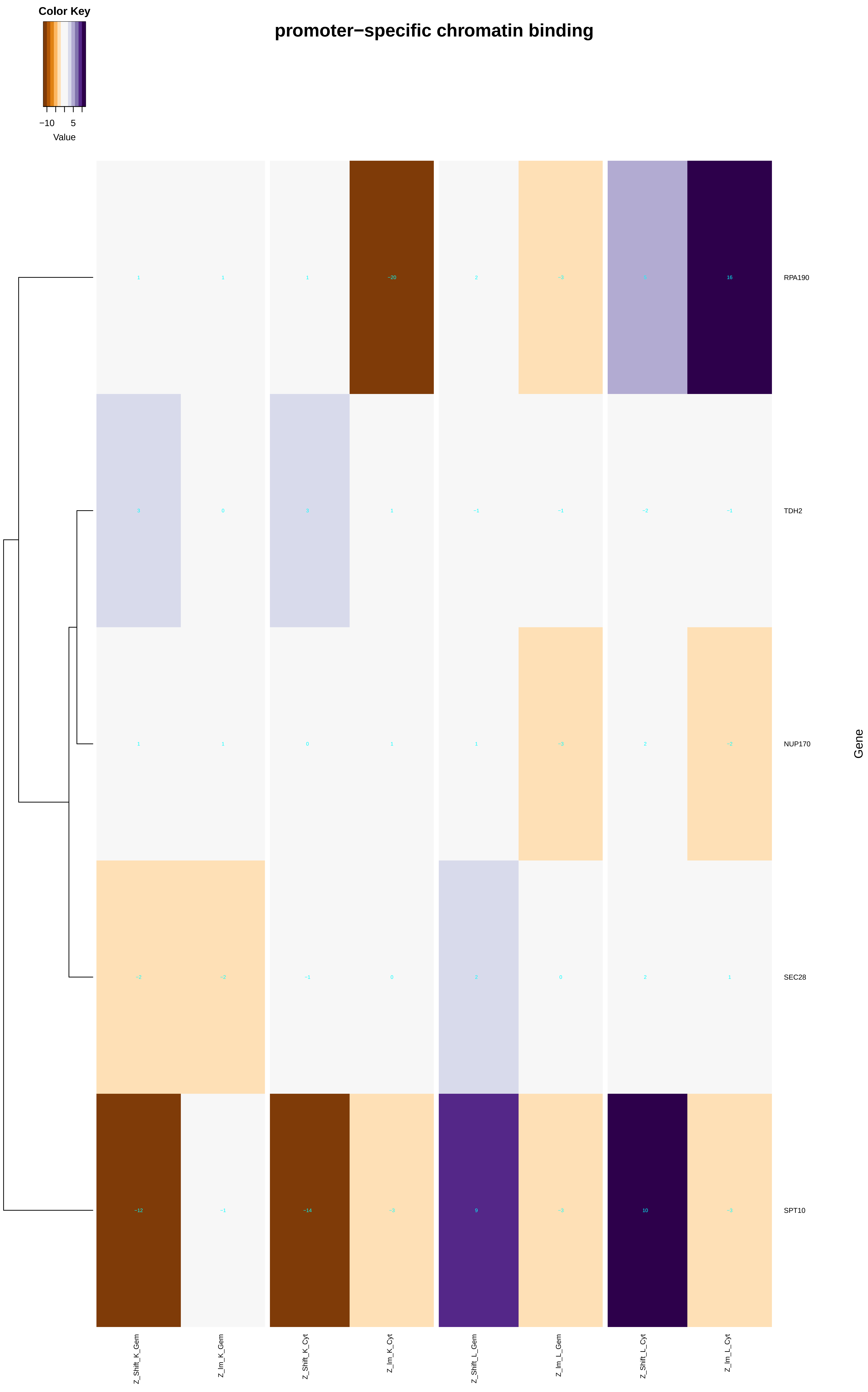
Color Key
−10
5
Value
promoter−specific chromatin binding
RPA190
TDH2
NUP170
SEC28
SPT10
Gene
Z_Shift_K_Gem
Z_Im_K_Gem
Z_Shift_K_Cyt
Z_Im_K_Cyt
Z_Shift_L_Gem
Z_Im_L_Gem
Z_Shift_L_Cyt
Z_Im_L_Cyt

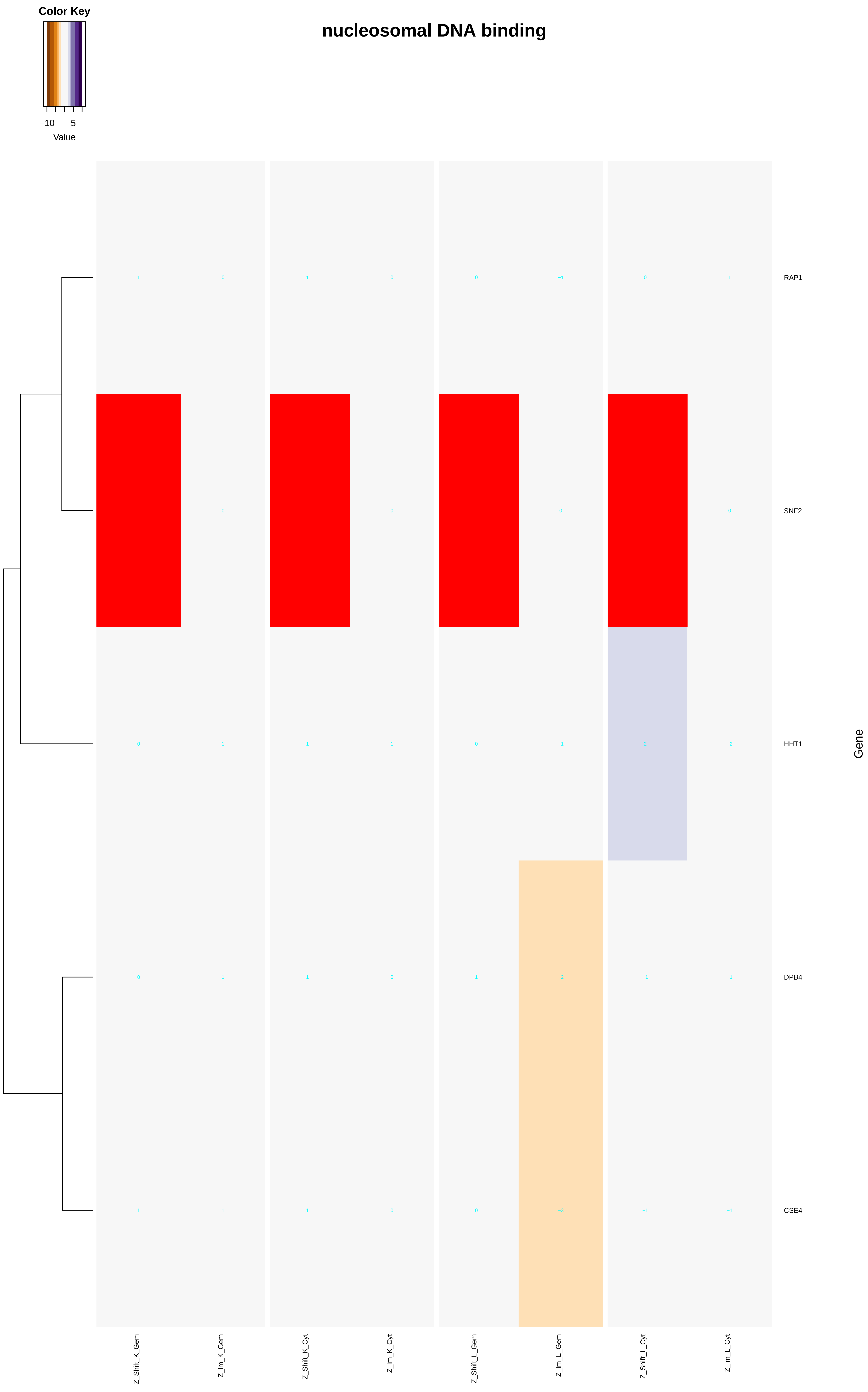

nucleosomal DNA binding
Color Key
−10
5
Value
RAP1
SNF2
HHT1
DPB4
CSE4
Gene
Z_Shift_K_Gem
Z_Im_K_Gem
Z_Shift_K_Cyt
Z_Im_K_Cyt
Z_Shift_L_Gem
Z_Im_L_Gem
Z_Shift_L_Cyt
Z_Im_L_Cyt

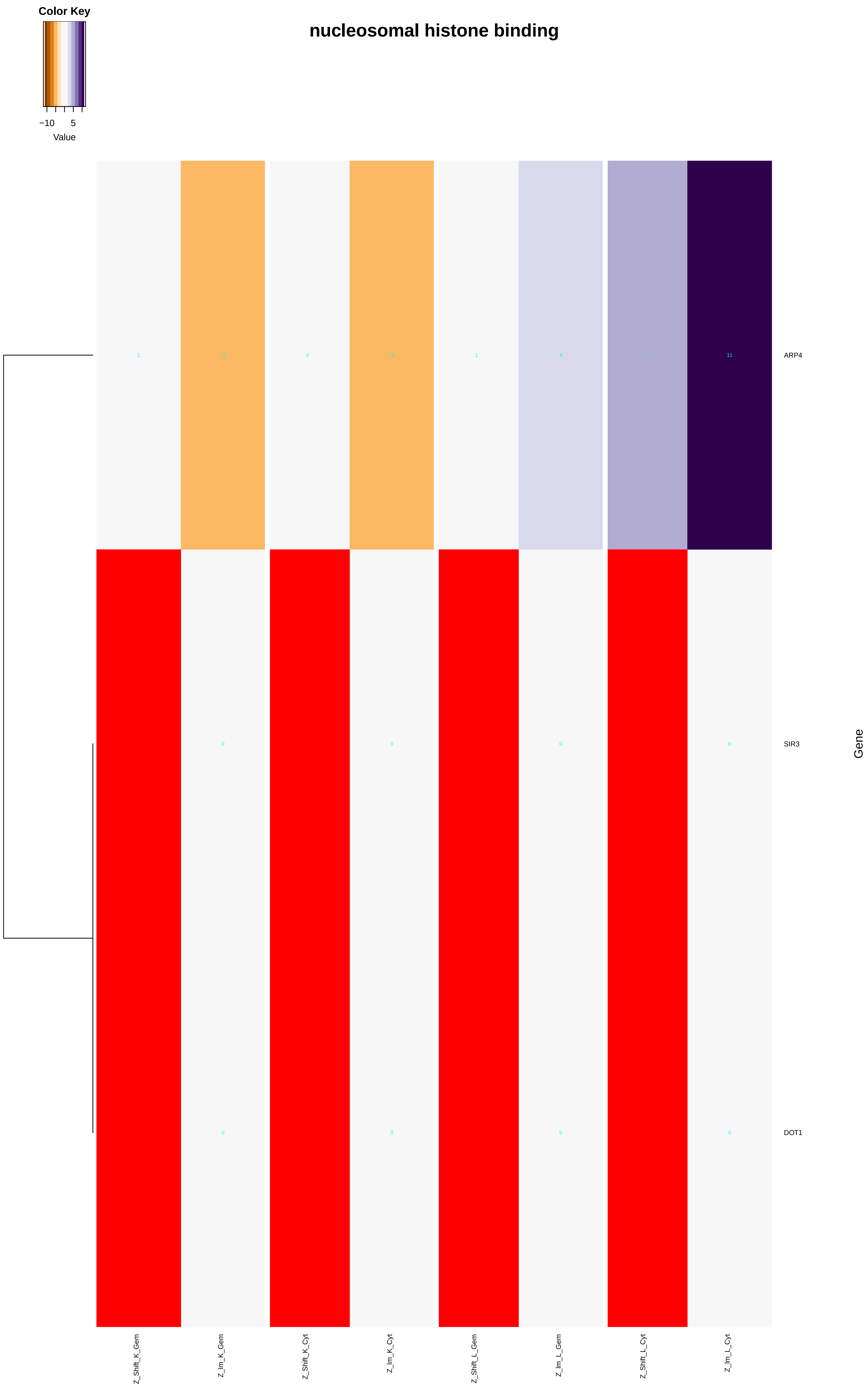

Color Key
−10
5
Value
nucleosomal histone binding
1
0
1
4
11
0
0
0
0
0
0
0
0
ARP4
SIR3
DOT1
Gene
Z_Shift_K_Gem
Z_Im_K_Gem
Z_Shift_K_Cyt
Z_Im_K_Cyt
Z_Shift_L_Gem
Z_Im_L_Gem
Z_Shift_L_Cyt
Z_Im_L_Cyt
